# Exploring Changes in Event-Related Potentials After a Feasibility Trial of Inhibitory Training for Bulimia Nervosa and Binge Eating Disorder

**DOI:** 10.3389/fpsyg.2020.01056

**Published:** 2020-05-27

**Authors:** Rayane Chami, Janet Treasure, Valentina Cardi, María Lozano-Madrid, Katharina Naomi Eichin, Grainne McLoughlin, Jens Blechert

**Affiliations:** ^1^Section of Eating Disorders, Department of Psychological Medicine, King’s College London, London, United Kingdom; ^2^Department of Psychiatry, Bellvitge University Hospital-Institut d’Investigacio Biomedica de Bellvitge (IDIBELL), Barcelona, Spain; ^3^CiberObn, Madrid, Spain; ^4^Department of Psychology, Centre for Cognitive Neuroscience, Paris-Lodron-University of Salzburg, Salzburg, Austria; ^5^Social, Genetic, and Developmental Psychiatry Department, Institute of Psychiatry, Psychology, and Neuroscience, King’s College London, London, United Kingdom

**Keywords:** event-relate potentials, binge eating disorder, bulimia nervesa, change process, ERPs

## Abstract

In a feasibility trial comparing two forms of combined inhibitory control training and goal planning (i.e., food-specific and general) among patients with bulimia nervosa (BN) and binge eating disorder (BED), we found evidence of symptomatic benefit, with stronger effects among participants receiving a food-specific intervention. The aim of the present study was to examine changes in behavioral outcomes and event-related potentials (ERPs; N2 and P3 amplitudes) from baseline to post-intervention that might suggest the mechanisms underpinning these effects. Fifty-five participants completed go/no-go tasks during two electroencephalography (EEG) sessions, at baseline and post-intervention. The go/no-go task included “go” cues to low energy-dense foods and non-foods, and “no-go” cues to high energy-dense foods and non-foods. Datasets with poor signal quality and/or outliers were excluded, leaving 48 participants (*N* = 24 BN; *N* = 24 BED) in the analyses. Participants allocated to the food-specific, compared to the general intervention group, showed significantly greater reductions in reaction time to low energy-dense foods, compared to non-foods, by post-intervention. Commission errors significantly increased from baseline to post-intervention, regardless of stimulus type (food vs. non-food) and intervention group (food-specific vs. general). There were no significant changes in omission errors. P3 amplitudes to “no-go” cues marginally, but non-significantly, decreased by post-intervention, but there was no significant interaction with stimulus type (high energy-dense food vs. non-food) or intervention group (food-specific vs. general). There were no significant changes in N2 amplitudes to “no-go” cues, N2 amplitudes to “go” cues, or P3 amplitudes to “go” cues from baseline to post-intervention. Training effects were only marginally captured by these event-related potentials. We discuss limitations to the task paradigm, including its two-choice nature, ease of completion, and validity, and give recommendations for future research exploring ERPs using inhibitory control paradigms.

## Introduction

### Rationale

The number of individuals receiving an eating disorder diagnosis has been increasing since the 1980s (Currin et al., 2005). This is particularly evident for binge eating disorder (BED), which has been increasing significantly in the new millennium (Micali et al., 2013). In order to improve the quality of current treatments for bulimia nervosa (BN) and BED it is essential to gain a better understanding of mechanisms that underpin binge-eating behavior.

Impulsivity is considered to be a risk factor for binge eating (Nasser et al., 2004). Reviews of cross-sectional research indicate that individuals with BED (Leombruni et al., 2014; Wu et al., 2016) and BN (Waxman, 2009; Vaz-Leal et al., 2015) show increased general (trait) impulsivity and eating-related impulsivity (Schag et al., 2013, 2019; Kessler et al., 2016; Giel et al., 2017). Furthermore, longitudinal studies conducted among individuals with BED have suggested that impulsivity is an impediment to treatment success (Meule and Platte, 2015; Manasse et al., 2017; Treasure et al., 2018).

As a multidimensional construct, impulsivity is thought to consist of two main components: decreased inhibitory control and increased reward sensitivity (Dawe and Loxton, 2004). Accordingly, impairments in inhibitory control have consistently been linked to increased eating disorder psychopathology (Svaldi et al., 2014; Manasse et al., 2016). Reward sensitivity, on the other hand, can be measured using behavioral tasks that explore implicit cognition, such as attentional biases (Deluchi et al., 2017). Stimuli that are highly motivationally relevant are likely to bias attention, in such a way where attention is directed toward a particular class of stimuli. Among individuals with binge eating behavior and/or obesity, attentional biases toward food cues, indicated by quicker reaction times to foods as opposed to non-foods during visual probe tasks, have been consistently reported (Castellanos et al., 2009; Nijs et al., 2010; Werthmann et al., 2011; Nijs and Franken, 2012; Jansen et al., 2015; Deluchi et al., 2017). Furthermore, this attentional bias may reflect difficulty disengaging from food stimuli, and greater reward while processing them (Leehr et al., 2018). It is hypothesized that this bias may, in turn, prevent individuals with binge eating behaviors from engaging in effective down-regulation of impulses toward food (Deluchi et al., 2017).

Event-related potentials (ERPs), derived from EEG recordings, offer the possibility of exploring cognitive processes within neural circuits (Luck, 2014). The N2, a negative fronto-central ERP observed ∼200–300 ms after stimulus presentation, has been used as a measure of inhibitory control and/or conflict monitoring (Falkenstein et al., 1999; Folstein et al., 2008; Watson and Garvey, 2013). The N2, localized to the anterior cingulate cortex (Lange et al., 1998; Liotti et al., 2000) is thought to reflect inhibitory control because it is enhanced to “no-go” compared to “go” stimuli (Enriquez-Geppert et al., 2010). In food-related tasks, N2 amplitudes are more negative when participants with binge eating behaviors are asked to inhibit to food, as opposed to non-food stimuli (Wolz et al., 2017) and this is particularly relevant to high energy-dense, as opposed to low-energy dense foods (Carbine et al., 2018). Nonetheless, there is uncertainty as to whether enhanced activation of N2 in response to high energy-dense food is a specific feature of binge-type eating disorders (Leehr et al., 2018; Chami et al., 2019), as it has also been reported among individuals in the higher BMI ranges (Carbine et al., 2018).

The P3 is an ERP with a positive peak that is elicited ∼300–600 ms after stimulus presentation (Albert et al., 2013). Its functional significance varies depending on the task at hand and it can reflect various cognitive processes, including target identification (Luck, 2014), working memory/context updating (Carbine et al., 2018), motivated attention (Schienle et al., 2008), or inhibitory control (Blackburne et al., 2016). Given that P3 responses are elicited in response to several cognitive processes, several variants have been described (see Polich, 2007 for an in-depth review). For instance, the P3a is often enhanced within fronto-central electrodes, and its generators are localized in cingulate, frontal, and right parietal areas (Volpe et al., 2007). It has been particularly relevant to inhibitory tasks (e.g., stroop task, stop-signal Tasks, or oddball paradigms; Polich, 2007; Blackburne et al., 2016). It has been thought to reflect a later stage that involves inhibition of the motor system (Dimoska et al., 2006), which may be particularly relevant to disinhibited eating behavior (i.e., binge eating; Smith et al., 2018). Exploring the *P3a* among participants with healthy, overweight, and obese BMIs, amplitudes were enhanced when the task involved inhibiting to high energy-dense, as opposed to low-energy dense foods (Carbine et al., 2018). In contrast, the *P3b* is more enhanced over parietal electrodes, and its generators are localized in bilateral, parietal, limbic, cingulate, and temporo-occipital regions (Volpe et al., 2007). It has been particularly relevant when exploring motivational relevance and salience (Herrmann et al., 2000). In line with this, several studies have reported enhanced P3b amplitudes toward food, as opposed to neutral non-food stimuli across all weight groups (Nijs et al., 2008, 2010; Hill et al., 2013; Hofmann et al., 2015). Due to the value of food for survival, food stimuli may represent natural “intrinsic targets,” even in the absence of specific experimental demands.

Recent evidence suggests that neural mechanisms underlying these executive functions can be trained, and that inhibitory control may be conceptualized as a muscle than can be strengthened with exercise (Benikos et al., 2013; Blackburne et al., 2016; Jones et al., 2016). Go/no-go training is one of the methods that has been used to train inhibitory control toward food cues (Lawrence et al., 2015; Allom et al., 2016; Jones et al., 2016). This training requires a rapid response to “go” stimuli, and inhibition to “no-go” stimuli (Lawrence et al., 2015). It is hypothesized that repeatedly pairing inhibitory responses to specific cues can strengthen the association between the cue and the behavioral goal (Houben and Jansen, 2011; Turton et al., 2016). This has been evidenced by several treatment trials, which have also found that using food-specific go/no-go trainings, as opposed to a general go/no-go tasks (i.e., with non-food stimuli) is more effective at decreasing unhealthy eating behaviors among individuals who overeat (Houben and Jansen, 2011; Veling et al., 2011; Lawrence et al., 2015).

To our knowledge, only one published study on disordered eating (Blackburne et al., 2016) has used ERPs as a means of assessing treatment outcomes. Within the study, participants with BMIs in the obese range who received food-specific inhibitory control training exhibited enhanced “no-go” P3 (i.e., P3a) amplitudes post-intervention, while those allocated to the waitlist control showed the opposite effect. The authors have interpreted this as an improvement in inhibitory control processing (Blackburne et al., 2016).

### Aims and Hypotheses

#### Aims

Recent evidence from a feasibility trial in our laboratory has found that an intervention combining go/no-go training and implementation intentions is associated with reductions in binge eating frequency among individuals with bulimia nervosa and BED (Chami et al., 2019). According to the Medical Research Council’s (MRC) guidelines, a key element of the development and evaluation process is to understand change processes underlying intervention efficacy (Craig and Petticrew, 2013). In line with this, the primary aim of the present research was to examine behavioral (i.e., reaction times, omission errors, and commission errors) and event-related potential (i.e., N2 and P3) changes from baseline to post-intervention. Within this study, an omission error was defined as an error during “go” trials (a “no-go” response when the task requires a “go” response) and a commission error was defined as an error during “no-go” trials (a “go” response when the task requires a “no-go” response). Moreover, the research aims to explore whether participants receiving a *food-specific intervention*, as opposed to a general intervention, would show additional changes in reaction time, omission errors, commission errors, N2, and P3 amplitudes in response to food cues from baseline to post-intervention. To explore the relationship between ERPs and core binge-type eating disorder symptomatology, the research also aims to explore correlations between changes in binge eating frequency (Chami et al., submitted) and changes in N2 and P3 amplitudes to high energy-dense foods. Since the timing and onset of N2 and P3 can vary as a function of processing speed and training, we explore their respective latencies too.

#### Hypotheses

##### Behavioral

We hypothesized that reaction time and omission errors to “go” cues (i.e., low energy-dense food and non-food) and commission errors to “no-go” cues (i.e., high energy-dense food and non-food) will decrease from baseline to post-intervention. These effects will be more pronounced for food cues, and among individuals receiving a food-specific intervention.

##### N2 and P3

In response to “no-go” cues, we hypothesized that mean “no-go” N2 amplitudes will increase from baseline to post-intervention, indicative of improved inhibitory control, and that mean “no-go” P3 amplitudes will decrease from baseline to post-intervention, indicative of reduced motivated attention. The opposite pattern is expected for “go” cues. Again, these effects will be more pronounced to food cues, and among individuals receiving a food-specific intervention. Finally, we predict that the training effects on binge eating will correlate with “no-go” N2 and P3 amplitudes to high energy-dense foods.

## Materials and Methods

### Participants

Participants with bulimia nervosa (*N* = 40) and BED (*N* = 38) were recruited through eating disorder charity websites, social media, flyers, and participant identification centers that supported the study. They were then randomly allocated to a food-specific or general intervention, which included both go/no-go training and goal planning (please refer to clinicaltrials.gov ID NCT03126526 for details of methodology; Chami et al., in submission). Within this manuscript, only participants who attended and completed both baseline and post-intervention EEG sessions were included (*N* = 55).

Eligibility required that participants met criteria for bulimia nervosa or BED according to the *Structured Clinical Interview for DSM-V*, had a Body Mass Index (BMI) of at least 18.5, were between the ages of 18 and 60, did not have a visual impairment that could not be repaired with eyewear, a neurological impairment, an alcohol or drug dependence, or psychosis.

### Assessment

#### Self-Report Measures

Eligibility clearance. The Structured Clinical Interview for DSM-V (SCID-5; First, 2014), a semi-structured interview for making a DSM-V diagnosis, was used to confirm diagnosis among participants with bulimia nervosa and BED, and to ensure no history of any psychiatric disorder among healthy control participants. All other eligibility criteria (i.e., age, neurological impairment, visual impairment, and BMI) were assessed with a short interview.

Binge eating frequency. Item 13 of the eating disorder examination questionnaire (EDE-Q; Fairburn, 2008) was used as a standalone outcome to assess binge eating frequency (*Over the last 28 days, how many times have you eaten what other people would regard as an unusually large amount of food?*).

#### Behavioral Measures

**FIGURE 1:**
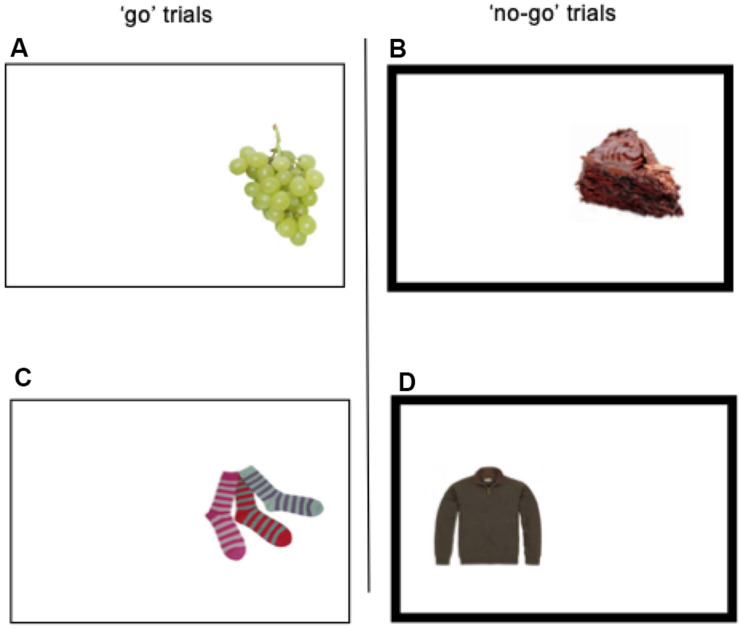
Picture **(A)** represents the presentation of a healthy food on the right side of the rectangle on screen. For this condition, participants were required to press the letter “m” as quickly as possible (“go” trial). The same applies to the condition picture **(C)**. Picture **(B)** represents the presentation of a palatable food on the right side of the rectangle on screen. For this condition, participants were required not to respond, because the border of the rectangle is bold (“no-go” trial). The same applies to the condition in picture **(D)**.

Food-specific go/no-go task. The present study used the food-specific go/no-go task, as implemented by Lawrence et al. (2015). During each trial within the task, one of 36 pictures was laterally presented (equiprobable on the left- or right- side) on a 19-inch computer screen for 1250 ms, with a 1250 ms inter-stimulus interval. Participants were seated at a 20-inch distance from the screen. The stimuli consisted of 9 low-energy dense food pictures (e.g., fruits, vegetables, and rice cakes), 9 high-energy dense foods food pictures (e.g., chocolate, cake, and crisps), and 18 non-food pictures (i.e., clothing items). Some of the food pictures had been previously used by fMRI studies of cue-reactivity, and they had been rated as pleasant (Beaver et al., 2006; Lawrence et al., 2012). A non-bold frame surrounding the picture and bold frame surrounding the picture, respectively, identified the “go” and “no-go” trials (see Figure 1). Non-bold frames remained on the screen during inter-trials. During “go” trials, participants were required to press “c” or “m” on the keyboard depending on the location of the picture on the screen (“c” for left and “m” for right). During the “no-go” trials, participants had to withhold their response. High-energy dense food pictures were always paired with “no-go” signals, resulting in 54 “no-go” trials, while the healthy food pictures were always paired with “go” signals, resulting in 54 go trials. The non-food pictures were equally likely to be paired with “go” and “no-go” frames. Each of the 36 pictures (9 + 9 + 18) was presented once per block, and participants completed 6 blocks per training session. The lack of “go” trials to high-energy dense food and “no-go” trials to healthy foods was due to the intervention that followed the session (Chami et al., in progress). Participants were provided with feedback regarding accuracy (error rate) and speed (mean reaction time) between blocks. Participants were instructed to respond as quickly and as accurately as possible. Food and non-food pictures were visually matched for size, color, and visual complexity (see Figure 1).

#### EEG

EEG was recorded continuously throughout the experimental tasks using BrainVision Recorder, and amplified with two 32-channel BrainAmp DC amplifiers (Brain Products GmbH, Munich, Germany) An actiCAP 64Ch standard cap was equipped after the 10–20 system (Jasper, 1958). FCz was used as the reference electrode, and AFz was used as the ground electrode. Impedances were kept below 15 KOhm for all the electrodes. Recording was performed with a sample rate of 500 Hz and an online bandpass filter between 0.1 and 100 Hz.

Offline, EEG data pre-processing was done using EEGLab (Delorme and Makeig, 2004) and comprised of the following steps: down-sampling to 256 Hz, manually removing bad channels, adding a zero channel and converting to average reference, high pass filtering at 1 Hz, which has been shown to be optimal for Independent Component Analysis (ICA), conducting ICA decomposition (AMICA; Palmer et al., 2012), identifying components for removal, extracting eye-blink, lateral eye movement, and facial muscle (e.g., jaw clenching) components, low pass filtering at 30 Hz, interpolating the removed channels, manually removing artifacts, segmenting the data into −500 ms pre-stimulus 1250 ms post-stimulus epochs, manually removing epochs with commission or omission errors, and baseline correcting (−500 ms–0 ms). Datasets from 4 participants were excluded from ERP analysis due to poor signal quality, leading to abnormal recordings. Moreover, 3 participants with N2 and P3 outliers were detected (Z > |3.0|) and case-wise excluded from all EEG analyses.

**FIGURE 2:**
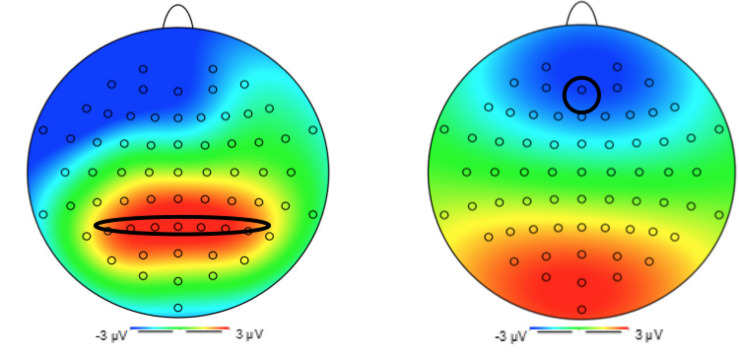
Topography 1: P3 at electrode Pz. Topography 2: N2 at electrode Fz.

Primarily driven by previous EEG studies using the go/no-go task (e.g., Carbine et al., 2018), we had planned to examine P3 amplitudes in fronto-central regions. In the present data, however, a P3-like local maxima was found over parietal electrodes between 300 and 600 ms. This finding indicated that, despite the inhibitory nature of the task we had adopted, the unchallenging nature of it may have led to a “salience-related” response, thus evoking P3b amplitudes (Polich, 2007). Our analyses of P3 amplitudes thus focused on attentional allocation and biases. P3 latencies were extracted as the time when the amplitude reached 50% of its peak amplitude. The electrodes that were identified for extraction were in the parietal region (P5, P3, P1, Pz, P2, P4, and P6; see Figure 2).

Similarly, based on previous literature (Carbine et al., 2018) and our topography (see Figure 2), N2 amplitudes and latencies were extracted from a frontal electrode (Fz). The N2 amplitude was extracted as the mean amplitude at electrode Fz occurring 200–350 ms post stimulus presentation, and the N2 latency was extracted as the time when the amplitude reached 50% of its peak.

### Procedure

Individuals who expressed interest in learning about the study procedures were sent an information sheet detailing the procedure. Next, they were contacted for a 15-min eligibility phone interview. Those who met criteria were sent a consent form indicating their rights as participants. After informed consent, an appointment was booked for the first EEG session and participants were sent a battery of questionnaires to complete via an online platform (i.e., Qualtrics, Provo, UT). Before entering the laboratory for the first EEG session, participants were instructed to withhold from food, caloric drinks, and nicotine for 2 h, as well as caffeinated drinks and alcohol for 24 h. During the laboratory session, participants were briefed about what the session will involve. They were then asked to sign a hard copy of the consent form, and their weight and head circumference were measured. After the appropriate EEG cap size was selected, the researcher put the cap on and applied electro-gel into the electrodes.

Before completing computerized tasks during EEG recording, participants were instructed to rest with their eyes open for 3 min and to rest with their eyes closed for 3 min. The researcher then explained the task rules and participants completed a practice trial of the go/no-go task, which included only 36 of the 216 trials of the full-length version. The average duration of the session was 2 h and 30 min. After 30 ± 2 days (i.e., post-intervention), the same procedure was followed for the second EEG session.

All procedures were revised and approved by the London Westminster Research Ethics Committee and the Health Research Authority (IRAS Project ID: 209609).

### Study Design

The study followed a mixed models design, with intervention (food-specific vs. general) as the between subject variable and time (baseline vs. post-intervention) as the between subject variables.

### Interventions

#### Inhibitory Control Training (Go/No-Go)

The inhibitory control training used was developed at the University of Exeter (Lawrence et al., 2015). Participants were encouraged to try to complete a computer-based go/no-go training task daily for 4 weeks. Participants allocated to the food-specific intervention group were asked to complete a food-specific go/no-task that is identical to the one described in section Implementation Intentions (If-Then Planning). Participants allocated to the general intervention group were asked to complete a general go/no-go task that had the same set of rules, but did not include food stimuli. While “go” and “no-go” trials were still present, the 18 food pictures were replaced with pictures of tools and stationery (see Lawrence et al., 2015 for details).

#### Implementation Intentions (If-Then Planning)

Implementation intentions involved encouraging participants to identify an unhelpful habit, reflect on situations and motivations that are likely to precede the unhelpful behavior, and then design an alternative behavior that could replace the unhelpful behavior. Participants allocated to the food-specific intervention group were asked to select an unhelpful behavior that was related to food/eating, while those allocated to the general intervention group were asked to select an unhelpful behavior that was unrelated to food/eating (e.g., social trouble). One example would be: “If I am home alone (situation) and feeling anxious (motivation), then I normally buy binge food (unhelpful eating-related habit),” would be replaced with “If I am home alone and feeling anxious, then I will meditate for 10 min (alternative behavior).” Each participant was assigned a trained mentor who followed up with him/her weekly via email for 4 weeks.

### Statistical Analysis

Statistical analyses were conducted using SPSS 24 (IBM Corp, 2016) for Mac. Primarily, descriptive and frequency statistics were used to report the mean and standard deviation of intervention engagement, while splitting for intervention group. An independent samples *t*-test was conducted to explore between-group differences in training task completion, and a chi-squared test was conduced to explore between group differences in implementation intention (i.e., goal planning) engagement. Next, independent samples *t-*tests were conducted to ensure that the two interventional groups did not significantly differ on demographic and clinical characteristics.

For behavioral data analysis, a repeated measures ANOVA was conducted to measure reaction time to “go” cues at two time points, across two types of stimuli, and between two intervention groups. The same ANOVA structure was used to analyze commission and omission errors, separately. ANOVAs followed the structure: 2 (time: pre- vs. post- intervention) × 2 (type of stimulus: low/high energy-dense food vs. non-food) × 2 (intervention group: food-specific vs. general intervention). “No-go” analyses included high energy-dense foods and non-foods, while “go” analyses included low energy-dense foods and non-foods.

To ensure that ERP amplitudes reflected the expected task demands, two paired samples *t*-tests were used to compare N2 and P3 amplitudes to “no-go” and “go” non-food cues.

For the main analysis, two repeated measures ANOVAs (for P3 and N2 separately) were conducted to measure amplitudes to “no-go” cues at two time points, across two types of stimuli, and between two intervention groups. They followed the format: 2 (time: pre- vs. post- intervention) × 2 (type of stimulus: high energy-dense food vs. non-food) × 2 (intervention group: food-specific vs. general intervention). This was repeated for “go” cues, with the following format: 2 (time: pre- vs. post- intervention) × 2 (type of stimulus: low energy-dense food vs. non-food) × 2 (intervention group: food-specific vs. general intervention). The main analyses were repeated for ERP latencies.

Finally, two-tailed Pearson’s correlations were used to assess the correlation between changes in binge eating frequency and changes in ERP amplitudes to high energy-dense foods across time. The variables were created using the following formulas: (1) Baseline *minus* post-intervention no-go N2 amplitudes to high energy-dense food, (2) Baseline *minus* post-intervention no-go P3 amplitudes to high energy-dense food, and (3) Baseline *minus* post-intervention binge eating frequency. This analysis structure was repeated for ERP latencies.

## Results

### Participant Characteristics

No significant differences in demographic and clinical characteristics were found between the two intervention groups (all *p* > 0.05; see Table 1). The average number of go/no-go training tasks completed was 13.50 (out of 28 total trainings; *SD* = 6.79). There was no significant difference in the number of trainings completed between participants in the food-specific intervention group (*M* = 14.64; *SD* = 6.42) and participants in the general intervention group [*M* = 12.26; *SD* = 7.10; *t*(46) = 1.22, *p* = 0.229]. With regards to if-then planning, 50% of participants were minimally engaged at implementing their plan. There were no significant differences in engagement between participants in the food-specific vs. general intervention group [*X*^2^(3, 48) = 5.247, *p* = 0.155].

**TABLE 1:** Baseline demographic and clinical characteristics of the sample.

	**Food-specific intervention**	**General intervention**	*p*-value*

	**(*N* = 25)**	**(*N* = 23)**	
	**M (*SD*) or *N* (%)**	**M (*SD*) or *N* (%)**	
**Demographic characteristics**			
Age	38.36 (12.03)	34.78 (13.32)	0.33
Weight (kg)	83.28 (23.49)	74.29 (24.66)	0.21
BMI	29.77 (6.87)	26.36 (8.33)	0.13
Duration of illness (Years)	19.10 (14.41)	16.74 (11.11)	0.57
Gender	Female = 21 (87.5%)	Female = 21 (91.3%)	0.67
	Male = 3 (12.5%)	Male = 2 (8.7%)	
Ethnicity	White = 19 (79.2%)	White = 17 (73.9%)	0.11
	Black = 1 (4.2%)	Black = 1 (4.3%)	
	Middle eastern = 3 (12.5%)	Mixed (White/Black) = 2 (8.7%)	
	Latin American = 1 (4.2%)	Asian = 3 (13%)	
**Clinical characteristics**			
Diagnosis	Binge eating disorder = 13 (52%)	Binge eating disorder = 11 (47.8%)	0.77
	Bulimia nervosa = 12 (48%)	Bulimia nervosa = 12 (52.2%)	
Comorbid mood and/or anxiety disorder	Yes = 20 (80%)	Yes = 19 (82.6%)	0.82
	No = 5 (20%)	No = 4 (17.4%)	
Use of psychiatric medication	Medication = 8 (33.3%)	Medication = 9 (39.1%)	0.68
	No medication = 16 (66.7%)	No medication = 14 (60.9%)	

*P-values for Age, Weight, BMI, and Duration of Illness were obtained using independent samples t-tests. P-values for Gender and Ethnicity using Fisher’s Exact Test. P-values for Diagnosis, Use of psychiatric medication, and Comorbid mood and/or anxiety disorder were obtained using Pearson’s Chi-Square.

### Behavioral Results

There was a significant main effect of time on reaction time [*F*(1, 46) = 28.12, *p* < 0.001, η^2^ = 0.379] and a significant interaction between time x type of stimulus x intervention group [*F*(1, 46) = 7.27, *p* = 0.01, η^2^ = 0.136], indicating that participants in the food-specific intervention, compared to the general intervention group, showed a significantly greater reduction (training effect) in reaction time to low energy-dense foods, compared to non-foods, by post-intervention (see Table 2 below). However, there were no significant main effects or interaction effects in omission errors (all *p* > 0.05). Although there was a main effect of time on commission errors [*F*(1, 46) = 12.78, *p* = 0.001, η^2^ = 0.217], the direction, indicating that participants made more errors by post-intervention, compared to baseline, was unexpected. No other significant main effects of interaction effects were significant (all *p* > 0.05) see Table 2 below.

**TABLE 2:** Mean reaction times and omission errors to low energy-dense foods and non-foods at baseline and post-intervention, and mean commission errors to high energy-dense foods and non-foods at baseline and post-intervention, split by intervention group.

		**Baseline M (*SD*)**	**Post-intervention M (*SD*)**	**Mean differences (95% CI)**	**Effect size (d_z_)**
RT low ED foods (ms)	Food-specific intervention	591.18 (91.32)	522.06 (84.50)	69.12 (38.01–100.24)	0.92
	General intervention	583.12 (121.52)	539.20 (114.98)	43.92 (8.19–79.65)	0.53
RT non-foods (ms)	Food-specific intervention	615.76 (99.37)	549.11 (92.04)	66.65 (32.94–100.36)	0.82
	General intervention	607.06 (126.27)	541.38 (103.64)	65.68 (28.17–103.18)	0.76
Omission error low ED foods	Food-specific intervention	1.64 (2.64)	1.56 (2.37)	0.07 (–1.32–1.46)	0.02
	General intervention	1.62 (4.23)	1.14 (2.08)	0.48 (–1.51–2.48)	0.10
Omission error non-foods	Food-specific intervention	2.23 (2.92)	1.05 (1.78)	1.19 (–0.24–2.62)	0.34
	General intervention	2.98 (5.08)	1.62 (2.47)	1.36 (–0.80–3.53)	0.27
Commission error high ED foods	Food-specific intervention	1.42 (3.09)	1.79 (2.90)	–0.37 (–1.78–1.04)	0.11
	General intervention	1.22 (1.75)	2.43 (2.76)	–1.20 (–2.40–0.004)	0.36
Commission error non-foods	Food-specific intervention	1.04 (1.70)	2.46 (2.66)	–1.42 (–2.48–0.35)	0.55
	General intervention	1.06 (1.57)	3.15 (3.13)	–2.09 (–3.60–0.58)	0.49

**ED, energy dense; N food-specific intervention, 25; N general intervention, 23; d_*z*_, effect size calculated for within subject power analyses.**

### Manipulation Check: Inhibition Evoked by Go/No-Go Task

**FIGURE 3:**
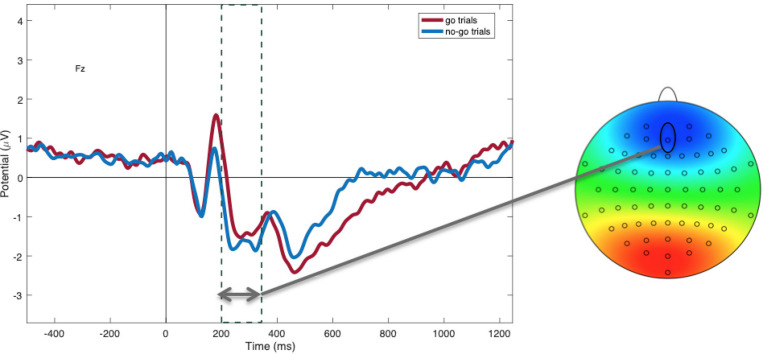
Mean N2 amplitudes at electrode Fz between 200 and 350 ms, showing more negativity during “no-go” trials (blue) compared to “go” trials (red; *p* = 0.01).

**FIGURE 4:**
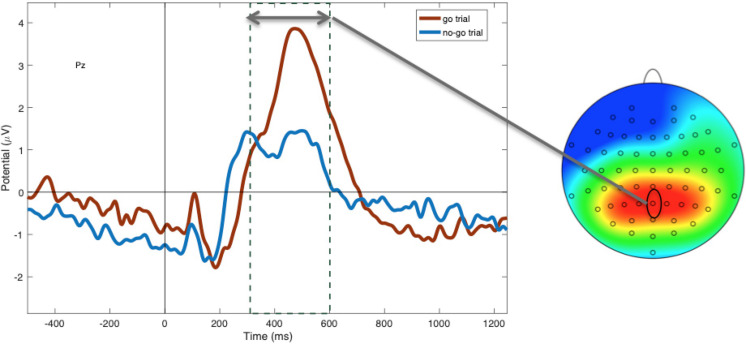
Mean P3 amplitudes at electrode Pz between 300 and 600 ms, showing more positivity during “go” (red) compared to “no-go” trials (blue; *p* < 0.001).

Within non-food trials, as predicted, N2 amplitudes were more negative to “no-go” (*M* = −1.45; *SD* = 1.73) compared to “go” cues [*M* = −0.87; *SD* = 2.03; *t*(48) = −2.62, *p* = 0.01; see Figure 3], suggesting greater inhibitory control in this condition. P3 amplitudes to non-foods were more positive to “go” cues (*M* = 1.99; *SD* = 0.29) compared to “no-go” cues [*M* = 1.48; *SD* = 0.21; *t*(47) = −4.20, *p* < 0.001; see Figure 4], suggesting that attention was enhanced during “go” trials and blunted during “no-go” trials.

### EEG Results

#### “No-Go” Cues

There was no main effect of time on “no-go” N2 amplitudes [*F*_(1,46)_ = 1.849, *p* = 0.181, η^2^ = 0.039]. Neither was there a time × type of stimulus × intervention group interaction effect [*F*(1, 46) = 0.014, *p* = 0.906, η^2^ = 0.001]. There was no significant main effect of time [*F*(1, 46) = 0.013, *p* = 0.911, η^2^ = 0.001] and no time x type of stimulus x intervention group interaction effect [*F*(1, 46) = 0.863, *p* = 0.358, η^2^ = 0.018] on “no-go” N2 latency.

There was a marginal, but non-significant, main effect of time on “no-go” P3 amplitudes [*F*(1, 46) = 3.801, *p* = 0.057, η^2^ = 0.076], but no significant time x type of stimulus x intervention group interaction [*F*(1, 46) = 0.015, *p* = 0.904, η^2^ = 001]. There was a significant main effect of time on “no-go” P3 latency [*F*(1, 46) = 12.47, *p* = 0.001, η^2^ = 0.213], indicating that “no-go” P3 latency decreased from baseline to post-intervention regardless of stimulus type. There was no time x type of stimulus x intervention group interaction effect [*F*(1, 46) = 0.010, *p* = 0.922, η^2^ = 0.001] on “no-go” P3 latency.

See Table 3 below for within group effect size calculations of no-go cues split by intervention group.

**TABLE 3:** N2 and P3 amplitudes and latencies to “No-Go” cues.

			**Baseline M (*SD*)**	**Post-intervention M (*SD*)**	**Mean differences (95% CI)**	**Effect size (d_z_)**
N2 high ED foods	Amplitude (μV)	Food-specific intervention	−1.75(2.17)	−1.92(1.51)	0.17 (−1.03–1.37)	0.06
		General intervention	−1.91(2.27)	−2.24(2.14)	0.34 (−0.93–1.61)	0.11
	Latency (ms)	Food-specific intervention	268 (37)	268 (31)	0.63 (−19.66–20.91)	0.01
		General intervention	285 (28)	288 (29)	−3.23 (−20.61–14.15)	0.10
N2 non-foods	Amplitude (μV)	Food-specific intervention	−1.29(1.70)	−1.93(2.41)	0.64 (−0.72–2.00)	0.19
		General intervention	−1.64(1.78)	−2.54(2.30)	0.90 (−0.29–2.10)	0.33
	Latency (ms)	Food-specific intervention	269 (24)	272 (31)	−3.13 (−15.42–9.18)	0.08
		General intervention	283 (26)	275 (33)	7.81 (−12.69–28.32)	0.16
P3 high ED foods	Amplitude (μV)	Food-specific intervention	1.68 (1.33)	1.74 (0.80)	−0.06 (−0.62–0.50)	0.04
		General intervention	2.20 (1.57)	1.75 (1.58)	0.45 (−0.10–1.01)	0.35
	Latency (ms)	Food-specific intervention	444 (41)	419 (49)	25.47 (−2.63–53.57)	0.37
		General intervention	448 (39)	425 (61)	22.08 (0.73–43.43)	0.45
P3 Non-foods	Amplitude (μV)	Food-specific intervention	1.22 (1.15)	1.16 (1.10)	0.06 (−0.53–0.65)	0.04
		General intervention	1.59 (1.78)	0.96 (1.53)	0.64 (0.09–1.19)	0.50
	Latency (ms)	Food-specific intervention	431 (52)	401 (62)	29.84 (0.69–59.00)	0.42
		General intervention	445 (34)	416 (69)	28.54 (1.43–55.63)	0.46

**ED, energy dense; μV, microvolts; N food-specific intervention, 25; N general intervention, 23; d_*z*_, effect size calculated for within group power analyses.**

#### “Go” Cues

There was no main effect of time on “go” N2 amplitudes [*F*(1, 46) = 1.849, *p* = 0.104, η^2^ = 0.056]. Moreover, the expected 3-way interaction was not significant [*F*(1, 46) = 1.536, *p* = 0.222, η^2^ = 0.032]. There was no significant main effect of time [*F*(1, 46) = 0.704, *p* = 0.406, η^2^ = 0.015] and no time × type of stimulus × intervention group interaction effect [*F*(1, 46) = 0.255, *p* = 0.616, η^2^ = 0.006] on “go” N2 latency.

There was no main effect of time on “go” P3 amplitudes [*F*(1, 46) = 0.678, *p* = 0.415, η^2^ = 0.015], and the expected 3-way interaction was not significant [*F*(1, 46) = 0.730, *p* = 0.397, η^2^ = 0.016]. There was a significant main effect of time on “go” P3 latency [*F*(1, 46) = 13.421, *p* = 0.001, η^2^ = 0.226], indicating that “no-go” P3 latency decreased from baseline to post-intervention regardless of stimulus type. There was no time x type of stimulus x intervention group interaction effect [*F*(1, 46) = 0.439, *p* = 0.511, η^2^ = 0.009] on “go” P3 latency.

See Table 4 below for within group effect size calculations of go-cues split by intervention group.

**TABLE 4:** N2 and P3 amplitudes and latencies to “Go” cues.

			**Baseline M (*SD*)**	**Post-intervention M (*SD*)**	**Mean differences (95% CI)**	**Effect size (d_z_)**
N2 low ED foods	Amplitude (μV)	Food-specific intervention	−1.18(1.94)	−1.96(2.20)	0.77 (−0.43–1.98)	0.26
		General intervention	−1.38(2.31)	−1.64(2.52)	0.26 (−1.13–1.65)	0.08
	Latency (ms)	Food-specific intervention	264 (39)	269 (35)	−5.16 (−24.72–14.31)	0.11
		General intervention	292 (30)	283 (41)	9.34 (−13.06–31.74)	0.18
N2 non-foods	Amplitude (μV)	Food-specific intervention	−0.96(1.89)	−1.64(2.53)	0.68 (−0.75–2.11)	0.20
		General intervention	−0.77(2.22)	−1.81(2.40)	1.05 (−0.19–2.28)	0.37
	Latency (ms)	Food-specific intervention	266 (36)	253 (29)	3.75 (−12.83–20.33)	0.09
		General intervention	274 (36)	264 (36)	9.68 (−10.88–30.24)	0.20
P3 low ED foods	Amplitude (μV)	Food-specific intervention	2.41 (1.78)	2.62 (1.30)	−0.20 (−0.77–0.36)	0.15
		General intervention	3.37 (2.45)	2.75 (2.10)	0.62 (−0.07–1.31)	0.39
	Latency (ms)	Food-specific intervention	446 (52)	425 (54)	20.47 (−2.42–43.36)	0.37
		General intervention	457 (43)	435 (45)	21.91 (0.69–43.13)	0.45
P3 non-foods	Amplitude (μV)	Food-specific intervention	1.90 (1.64)	2.07 (1.32)	−0.16 (−0.62–0.29)	0.15
		General intervention	2.46 (2.32)	2.09 (1.64)	0.37 (−0.34–1.08)	0.23
	Latency (ms)	Food-specific intervention	461 (56)	423 (41)	37.19 (7.15–67.22)	0.51
		General intervention	451 (48)	425 (48)	25.48 (−0.80–51.75)	0.42

**ED, energy dense; μV, microvolts; N food-specific intervention, 25; N general intervention, 23; d_*z*_, effect size calculated for within subject power analyses.**

#### Correlations With Changes in Binge Eating Frequency

Across all participants, the mean reduction in binge eating frequency was 3.95 (*SD* = 10.28).

There was no significant correlation between changes in binge eating frequency and changes in no-go N2 amplitude to high energy-dense foods (*r* = −0.139, *p* = 0.368) or N2 latency to high energy-dense food (*r* = −0.151, *p* = 0.326). Moreover, there was no significant correlation between changes in binge eating frequency and changes in no-go P3 amplitude to high-energy dense food (*r* = −0.284, *p* = 0.062) or P3 latency to high energy-dense food (*r* = −0.178, *p* = 247).

## Discussion

This study examined behavioral (i.e., reaction times, omission errors, and commission errors) and event-related potential (i.e., N2 and P3) changes at baseline and at the end of an intervention designed to modify inhibitory control for bulimia nervosa and BED. The research aimed to explore whether changes would be present, whether they would be specific to food, and whether they would differ between the two intervention groups.

In line with our hypothesis, individuals allocated to the food-specific intervention group, compared to the general intervention group, showed significantly greater reductions in reaction time to low-energy dense foods, compared to non-foods, from baseline to post-intervention. These indicate that successful stimulus-response learning to “go” cues had taken place, which may have induced a beneficial attentional bias toward these foods. Participants in both intervention groups showed significant reductions in P3 latency over time, indicating a speeding of task-related information processing (Kieffaber and Hetrick, 2005; Schaefer and Nooner, 2018). Contrary to our hypothesis, no significant changes in the number of omission errors were found. Furthermore, the number of commission errors increased from baseline to post-intervention, which was unexpected. This increase in commission errors may represent a speed-accuracy trade-off, where speeded reaction time is parallel to an increase in errors. It may also result from boredom or fatigue, as participants who complete the same training task, with no variation to interval durations, may have become more distractible.

At baseline, “no-go” cues elicited larger N2 amplitudes and smaller P3 amplitudes compared to “go” cues, suggesting that there was greater inhibition and less attention/salience toward “no-go” cues, lending evidence to the fundamental validity of the task and training.

Despite this, no significant increases in “no-go” N2 amplitudes were found from baseline to post-intervention, and no significant differences were found between the two intervention groups. This was in line with findings from previous research, which reported no increase in N2 amplitude as a result of inhibitory control training (Blackburne et al., 2016). Such findings, in addition to the non-significant correlation between changes in binge eating frequency and changes in “no-go” N2 to high energy-dense food may confirm that the N2 component might be more closely related to conflict monitoring (Dimoska et al., 2006), a process not targeted by the present interventions (i.e., inhibitory control training and implementation intentions). Previous research using a go/no-go task suggest that a greater number of “go,” as opposed to “no-go,” trials (i.e., where the stopping/no-go process needs to be evoked against a dominant, frequent response) are needed to evoke enhanced N2 amplitudes (Donkers and Van Boxtel, 2004).

Albeit marginally and non-significantly, P3 amplitudes to “no-go” cues descriptively decreased from baseline to post-intervention. Nonetheless, there was no significant interaction between time x type of stimulus x intervention group, and no significant correlation between changes in binge eating frequency and no-go P3 amplitudes to high energy-dense foods. These indicate that the marginal decrease in P3 to “no-go” cues may reflect a general depreciation that comes with repeated task completion. Given that the P3 amplitude is sensitive to the amount of attentional resources engaged (Polich, 2007), it is likely to reduce after the task is learnt over repeated trainings.

Our hypotheses regarding changes in N2 to “go” cues were not supported and no significant 3-way interaction was found. In addition, while we expected less negativity over time (suggestive of reduced inhibition), there was a non-significant trend in the opposite direction. These findings might have been influenced by the inclusion of a binary “left-right” decision to the “go” instruction (participants were required to press “C” or “M” depending on the location of the stimulus on the screen during “go” trials). This additional attentiontal control may have slowed down the “go” process and engaged inhibitory processes.

In addition, no significant changes in P3 amplitudes to “go” cues were found from baseline to post-intervention, and no significant 3-way interaction was found. While previous research using similar methodology had found enhanced P3 amplitudes in frontal electrodes over the course of training (Blackburne et al., 2016), the go/no-go task used in the present research failed to provoke a P3 response in frontal electrodes. Given that P3 amplitudes in the present study were more enhanced within parietal electrodes, which are thought to represent motivational relevance and salience (Herrmann and Knight, 2001; Heinze et al., 2007), attentional, as opposed to inhibitory, processes may have been involved.

In light of these findings, we were unable to identify changes in neural components that correlate with changes in eating behavior over time. Therefore, while changes in binge eating frequency were found on a behavioral level (Chami et al., in submission), it remains unclear what change processes had occurred on a neural level. A consideration of limitations is essential. For instance, the negligible error rates during task completion may suggest that the task was not challenging and hence, did not recruit inhibitory circuits. This is consistent with the absence of a P3 in fronto-central electrodes (Polich, 2007). To increase the differentiation between “go” and “no-go” trials, it may have been more informative to use a simple food go/no-go task, as opposed to a two-choice go/no-go task. Additionally, a random inter-trial interval may have increased our ability to ensure participants” attentiveness to the task. Another limitation of this study is that it combined inhibitory control training and if-then planning, making it challenging to assess the individual impact of each intervention. Moreover, while we used the go/no-go training task as a measure of change to allow for an understanding of what occurs during training completion, it has inevitably been designed as a training tool (Lawrence et al., 2015). Therefore, given that low energy-dense foods were always paired with “go” cues and high energy-dense foods were always paired with “no-go” cues, our ability to compare changes in ERP responses to high and low-energy dense foods over time was limited. These comparisons may be particularly relevant in individuals with eating disorders (see Carbine et al., 2018).

## Conclusion

The present research shows that, while participants in the intervention showed reductions in binge eating frequency (Chami et al., in submission), the neural processes supporting this clinical effect could not be entirely uncovered. It remains unclear whether the null findings reflect an absence of change in neural activity over time, or an inability of the measures to detect change. It is advisable for future research to explore different task parameters, by potentially differing the ratio of “go” to “no-go” trials and increasing the speed-accuracy trade-off.

## Data Availability Statement

The datasets generated for this study are available on request to the corresponding author.

## Ethics Statement

The London Westminster Research Ethics Committee and the Health Research Authority reviewed and approved all the procedures involved in this study (IRAS Project ID: 209609). The patients/participants provided their written informed consent to participate in this study.

## Author Contributions

RC has recruited participants and collected the data. GM has offered use of EEG equipment. RC and ML-M have pre-processed the data. KE has contributed to epoching the EEG data. JB has assisted in the creation of figures. RC has written the manuscript, with suggestions and corrections from JT, VC, ML-M, KE, GM, and JB.

## Conflict of Interest

ML-M was employed by company CiberObn. The remaining authors declare that the research was conducted in the absence of any commercial or financial relationships that could be construed as a potential conflict of interest.
